# Association between parental absence during childhood and metabolic syndrome during adulthood: A cross-sectional study in rural Khanh Hoa, Vietnam

**DOI:** 10.1371/journal.pone.0282731

**Published:** 2023-03-09

**Authors:** Rachana Manandhar Shrestha, Tetsuya Mizoue, Thuy Thi Phuong Pham, Ami Fukunaga, Dong Van Hoang, Chau Que Nguyen, Danh Cong Phan, Masahiko Hachiya, Dong Van Huynh, Huy Xuan Le, Hung Thai Do, Yosuke Inoue

**Affiliations:** 1 Department of Epidemiology and Prevention, Center for Clinical Sciences, National Center for Global Health and Medicine, Tokyo, Japan; 2 Department of Non-communicable Disease Control and Nutrition, Pasteur Institute in Nha Trang, Khanh Hoa, Vietnam; 3 Bureau of International Health Cooperation, National Center for Global Health and Medicine, Tokyo, Japan; 4 Khanh Hoa Center for Disease Control, Khanh Hoa, Vietnam; 5 Pasteur Institute in Nha Trang, Khanh Hoa, Vietnam; Shahid Beheshti University of Medical Sciences, School of Dentistry, ISLAMIC REPUBLIC OF IRAN

## Abstract

**Background:**

This study aimed to determine the association between parental absence during childhood and metabolic syndrome (MetS) in adulthood among middle-aged adults in rural Khanh Hoa province, Vietnam. Given that broader literature on adverse childhood experiences (ACEs) suggests a strong positive association between ACEs and cardiometabolic risk or diseases, we hypothesized that parental absence during childhood, which is a major component of ACEs, is more likely to cause MetS in adulthood.

**Methods:**

Data were obtained from the baseline survey of the Khanh Hoa Cardiovascular Study, in which 3000 residents aged between 40 to 60 years participated. MetS was assessed using the modified Adult Treatment Panel III (ATP III) criteria. It was considered parental absence if the participants had experienced parental absence due to death, divorce, or out-migration before three or between three to 15 years. We used multiple logistic regression analyses to examine the association between parental absence during childhood and metabolic syndrome during adulthood.

**Results:**

There was no significant association between parental absence and MetS; adjusted odds ratio [AOR] was 0.97 (95% confidence interval [CI] = 0.76–1.22) for those who experienced parental absence between three to 15 years and the corresponding figure for those who experienced it before three years was 0.93 (95% CI = 0.72–1.20). No significant associations were observed when these were examined for the causes of parental absence.

**Conclusion:**

This study did not support our hypothesis of an association between parental absence during childhood and metabolic syndrome during adulthood. Parental absence may not be a predictor of MetS among Vietnamese people in rural communities.

## Background

Parental absence in early life is traumatic and has long-lasting health effects [1, 2]. For example, a meta-analysis of nine studies from seven countries by Simbi et al. [3] reported that people who had experienced parental loss before 18 years of age were twice more likely to develop depression during adulthood. Previous studies also have suggested that parental loss is associated with other mental health outcomes including anxiety and schizophrenia [4–6].

Compared to mental health studies that have been extensively focused on parental absence during childhood, there is little research regarding parental absence on physical health during adulthood. Given that broader literature on adverse childhood experiences (ACEs) suggests a strong positive association between ACEs and cardiometabolic risk or diseases [7, 8], parental absence during childhood, a major component of ACEs, could cause a higher cardiometabolic risk in adulthood. A prospective study by Chen et al. [9] reported that parental loss during childhood tended to be associated with increased hypertension risk during adulthood. Moreover, a cross-sectional study of 135 Italian adult participants (mean age: 43.8 years old) by Alciati et al. [10] reported a significant association between parental loss during childhood and metabolic syndrome (MetS) in adulthood, although it was restricted to those with severe obesity (body mass index [BMI] ≥40 kg/m^2^).

This study was conducted to examine the association between parental absence during childhood and MetS in adulthood in Vietnam, where the prevalence of MetS was reported to range from 14.0% to 21.6% since 2014 to 2019 [11–13]. We believe Vietnam can be important research setting to examine the association for several reasons. First, many middle-aged adults might have lost or got separated from their parents during and after the Vietnam War (1955–1975). Previous studies have suggested that parental loss in childhood is associated with a worsening of cardiovascular health in adulthood [14–16]. Hence, those who experienced it during and after the war could be at a higher cardiometabolic risk. Second, the health impact of ACEs due to parental absence might have been exacerbated by lifestyle changes during the economic growth since the 1990s (e.g., westernization in diet). As suggested by the Developmental Origins of Health and Disease (DOHaD) hypothesis [17], the mismatch between the nutritional environment in childhood and that in adulthood might have contributed to the increase in the number of cardiovascular diseases (CVDs) [18, 19].

This study thus aimed to examine the association between parental absence during childhood and the prevalence of MetS in adulthood among the same study population. We hypothesized that those who experienced parental absence during childhood were more likely to have MetS in adulthood, and that this association would be stronger if they experienced it earlier in childhood (i.e., before the age of three) than later in childhood.

## Methods

### Study design and participants

Data for this study came from a baseline survey of the Khanh Hoa Cardiovascular Study (KHCS), which is an ongoing cohort study among 3000 rural residents which aimed at examining the determinants of CVD events, especially those specific to Vietnamese society. From a district in Khanh Hoa Province, eight communes, which were socio-economically average communities in rural Vietnam, were chosen as study sites.

The eligible participants for KHCS were those who were living in the study communes for more than six months and aged 40 to 59 years at the time of recruitment. Commune health center staff members prepared the lists of all eligible participants in each commune and recruited participants until the target number (i.e., 3000 participants) was reached (consent rate: 75–87%) (i.e., convenient sampling). Eligible participants were asked to participate in the survey in fasting state. Exclusion criteria for this study were: being unable to provide informed consent, who had a plan to move out of the community within one-year, pregnant women and those who gave birth within one year, those who had CVD events in the past, and those being institutionalized. The baseline survey of the KHCS was conducted between June 2019 and 2020. The detailed information of the survey is available in our previous studies by Inoue et al. [20] and Chau et al. [21].

The study procedure was approved by the Research Ethics Committee at NCGM (approval number: NCGM-G-003172-03), the Ethical Committee of the Pasteur Institute in Nha Trang, Vietnam (approval number: 02/2019/HDDD-IPN), and the Ethical Committee of the University of Tokyo (approval number: 2021007NI). Written informed consent was obtained from all participants.

### Anthropometric and biochemical measurements

Waist circumference was measured at the umbilical level in the standing position using a measuring tape. Systolic and diastolic blood pressures were measured twice with participants seated and their arms supported at the heart level using an electric sphygmomanometer (Omron, HEM1020, Tokyo, Japan). They were instructed to rest for at least five minutes before the first measurement. Two measurements were used to calculate the mean blood pressure. Plasma fasting glucose, high-density lipoprotein (HDL)-cholesterol, low-density lipoprotein (LDL)-cholesterol, and triglycerides were measured using a Cobas 8000 (Roche, Switzerland).

### Parental absence (exposure)

We considered it as parental absence if participants reported experiencing it before the age of three or between three and 15 years and due to the following causes: death, divorce, or out-migration for more than one year. Participants responded to the following questions: “Before you turned 15 years, did your parents die, get divorced or leave you for migratory work for a continuous period of more than one year?” and “Before you turned three years, did your parents die, get divorced or leave you for migratory work for a continuous period of more than one year?”. Response options included three categories (yes, no, and cannot recall), which were further grouped into two categories (no/cannot recall = 0 and yes = 1). We then categorized the participants into three categories: those who experienced parental absence before three years of age, those who experienced parental absence between three to 15 years, and those who did not have such experiences. To determine whether different causes of parental absence resulted in different associations, we also categorized participants according to parental absence experience by three different causes (i.e., death, divorce, and separation due to out-migration).

### Metabolic syndrome (outcome)

Metabolic syndrome was defined according to the modified National Cholesterol Education Program (NCEP) Adult Treatment Panel III (ATP III) criteria [22] and Asian-specific cut-offs were employed for waist circumference, which had been used in previous studies in Vietnam [11, 12]. Specifically, participants were classified as having MetS if they met three or more of the following five components: (1) fasting plasma glucose ≥ 5.6 mmol/L (100 mg/dL) or on anti-diabetic medication, (2) systolic blood pressure ≥ 130 mmHg or diastolic blood pressure ≥85 mmHg or on antihypertensive medication, (3) HDL-C <1.04 mmol/L (40 mg/dL) for men and HDL-C < 1.29 mmol/L (50 mg/dL) for women, (4) triglycerides ≥ 1.7 mmol/L (150 mg/dL) or on lipid-lowering medication, (5) and waist circumference ≥ 90 cm for men and ≥ 80 cm for women.

### Covariates

We collected information on the following socio-demographic information using a questionnaire: age (in years), sex (male or female), marital status (married or unmarried), educational attainment (less than primary school, primary school, middle school, or high school or higher), occupation (government employee, non-government employee, self-employed, farmer or fisherman, housewife, other, or not working), and household income (low, middle, or high). Information on monthly household income was obtained by asking the representatives to choose from the following categories in Vietnamese Dong (VND)(≤1,000,000; 1,000,001 to ≤ 2,000,000; 2,000,001 to ≤ 3,000,000; 3,000,001 to ≤ 4,000,000; 4,000,001 to ≤ 6,000,000; 6,000,001 to ≤ 8,000,000; 8,000,001 to ≤ 12,000,000; 12,000,001 to ≤ 16,000,000; ≤ 16,000,001 to ≤ 20,000,000; > 20,000,000; or do not know), which was divided by the square root of the number of household members (i.e., equivalized income) and then grouped into tertiles (low, middle, or high). Childhood socioeconomic status was defined using the response to “How would you rate your family’s socioeconomic status when you were 15 years according to standards of that time?”. Response options included five categories ranging from low to high, which we further categorized into (low, lower-middle, or middle/ middle-high/ high) due to the small sample size in the upper two categories.

We also collected information on the following health-related behaviors: smoking status (never, former, or current), and alcohol drinking (do not drink, less than one standard drink, 1–1.9 standard drink, or ≥2 standard drinks per day), and physical activity (calculated by total metabolic equivalent task) categorized into three groups (<600 METs, 600–1200 METs, or >1200 METs) based on the Global Physical Activity Questionnaire [23].

### Statistical analysis

Multiple imputations were used to account for those with missing information regarding household income (n = 33). Specifically, a multinomial logistic model was used to generate 20 datasets (200 iterations). The estimates were combined using Rubin’s rule [24].

The baseline characteristics of the participants, stratified by parental absence status, were expressed as means and standard deviations (SD) for continuous variables and numbers and proportions for categorical variables. To examine the association between parental absence by timing and causes and MetS, we conducted multiple logistic regression analyses to estimate odds ratios (ORs) and 95% confidence intervals (CIs) of MetS. In model 1, we adjusted for age and sex. We also adjusted for childhood socioeconomic status (i.e., a confounder in the association between parental absence and MetS) in model 2 (the main model). In model 3, we further adjusted for marital status, job category, education, household income, smoking, alcohol consumption, and physical activity as possible mediators linking parental absence and MetS. The statistical significance level was set at p < 0.05. All analyses were conducted using Stata ver.15.0 (College Station, TX, USA).

## Results

Table 1 shows the participants’ general characteristics according to parental absence status. Of the total participants, 636 (21.2%) had experienced parental absence before 15 years of age. The mean age of study participants was 49.9 years (SD = 5.5), 61.3% were female, and 89.3% were married, and the proportions were similar among those who had and had not experienced parental absence during their childhood. Participants who had parental absence experience before 3 years and between 3–15 years were more likely to have education level below primary school (15.6% and 17.3% vs. 10.5%), low socioeconomic status (45.3% and 51.0% vs. 37.0%), low household income (38.7% and 38.9% vs. 31.9%) and less likely to be a government employee (6.9% and 6.1% vs. 10.7%) compared to those who did not have such experience. The mean waist circumference, systolic and diastolic blood pressure, fasting blood glucose, high-density lipoprotein and triglycerides were similar between those who had and did not have such experience during their childhood.

**Table 1:** Characteristics of study participants in the Khanh Hoa Cardiovascular Study, Vietnam (2019–2020).

		Parental absence		
Variables	All participants	No	Yes (3 - <15 yo)	Yes (< 3 yo)
	(N = 3000)	(n = 2364)	(n = 347)	(n = 289)
Age (years), mean [SD]	49.9 [5.5]	49.6 [5.5]	50.9 [5.5]	50.6 [5.2]
Sex (female), n (%)	1,840 (61.3)	1,439 (60.9)	218 (62.8)	183 (63.3)
Ethnicity (Kinh), n (%)	2,970 (99.0)	2,337 (98.69)	346 (99.7)	287 (99.3)
Marital status (currently married), n (%)	2,680 (89.3)	2,125 (89.9)	304 (87.6)	251 (86.8)
Education, n (%)				
Less than primary school	352 (11.7)	247 (10.5)	60 (17.3)	45 (15.6)
Primary school	863 (28.8)	655 (27.7)	124 (35.7)	84 (29.1)
Middle school	1,068 (35.6)	862 (36.5)	108 (31.1)	98 (33.9)
High school or higher	717 (23.9)	600 (25.4)	55 (15.9)	62 (21.4)
Occupation, n (%)				
Government employee	295 (9.8)	254 (10.7)	21 (6.1)	20 (6.9)
Non-government employee	483 (16.1)	386 (16.3)	57 (16.4)	40 (13.8)
Self-employed	595 (19.8)	469 (19.8)	79 (22.8)	47 (16.3)
Farmer/fisherman	870 (29.0)	676 (28.6)	102 (29.4)	92 (31.8)
Houseworker	527 (17.6)	412 (17.4)	52 (15.0)	63 (21.8)
Other	111 (3.7)	85 (3.6)	15 (4.3)	11 (3.8)
Not working	119 (4.0)	82 (3.5)	21 (6.1)	16 (5.5)
Household income, n (%)				
Low	1002 (33.4)	755 (31.9)	135 (38.9)	112 (38.7)
Middle	1045 (34.8)	843 (35.7)	114 (32.9)	88 (30.5)
High	920 (30.7)	741 (31.3)	93 (26.8)	86 (29.8)
Missing	33 (1.1)	25 (1.1)	5 (1.4)	3 (1.0)
Childhood socioeconomic status, n (%)				
Low	1,183 (39.4)	875 (37.0)	177 (51.0)	131 (45.3)
Middle-low	764 (25.5)	593 (25.1)	88 (25.4)	83 (28.7)
Middle/ middle-high/ high	1,053 (35.1)	896 (37.9)	82 (23.6)	75 (26.0)
Smoking, n (%)				
Never	2,037 (67.9)	1,605 (67.8)	237 (68.3)	195 (67.5)
Former	349 (11.6)	273 (11.5)	43 (12.4)	33 (11.4)
Current	614 (20.5)	486 (20.6)	67 (19.3)	61 (21.1)
Alcohol consumption, n (%)				
Don’t drink	2,114 (70.5)	1,655 (70.0)	251 (72.3)	208 (72.0)
<1 standard drink	416 (13.9)	326 (13.8)	46 (13.3)	44 (15.2)
1–1.9 standard drink	201 (6.7)	165 (7.0)	24 (6.9)	12 (4.1)
≥2 standard drink	269 (8.9)	218 (9.2)	26 (7.5)	25 (8.7)
Physical activity (METs minutes/week), n (%)				
<600	252 (8.4)	201 (8.5)	25 (7.2)	26 (9.0)
600–1200	120 (4.0)	93 (3.9)	20 (5.8)	7 (2.4)
>1200	2628 (87.6)	2070 (87.6)	302 (87.0)	256 (88.6)
Waist circumference (cm), mean [SD]	81.1 [8.5]	81.1 [8.5]	80.6 [8.3]	81.1 [8.6]
Systolic blood pressure (mmHg), mean [SD]	130.9 [19.1]	130.8 [18.8]	131.4 [19.2]	131.1 [21.1]
Diastolic blood pressure (mmHg), mean [SD]	83.9 [12.1]	83.9 [12.0]	84.1 [12.5]	83.9 [13.2]
Fasting blood glucose (mmol/L), mean [SD]	5.5 [1.5]	5.5 [1.4]	5.6 [1.6]	5.7 [2.1]
High-density lipoprotein cholesterol (mmol/L), mean [SD]	1.3 [0.3]	1.3 [0.3]	1.3 [0.3]	1.3 [0.3]
Triglycerides (mmol/L), mean [SD]	2.0 [1.5]	2.0 [1.5]	1.9 [1.3]	1.9 [1.4]

SD, standard deviation; yo, years old

Table 2 depicts the results of multiple logistic regression analysis investigating the association between parental absence by the timing (before three years or between three and 15 years) and MetS. We did not find a significant association between parental absence and MetS among the study participants who had experienced parental absence between three and 15 years (e.g., model 2; AOR = 0.97, 95% CI = 0.76–1.22) and those who experienced it before three years (Model 2; AOR = 0.93, 95% CI = 0.72–1.20). The associations were statistically insignificant in model 3.

**Table 2:** Logistic regression analyses for the association between parental absence in childhood and metabolic syndrome in adulthood among study participants in the Khanh Hoa Cardiovascular Study, Vietnam (2019–2020).

Parental absence	Those with/without MetS	AOR	95% CI
**Model 1**			
No	887 / 1477	1.00	Ref.
Yes (3 - <15 yo)	133 / 214	0.96	0.76–1.21
Yes (<3 yo)	107 / 182	0.92	0.71–1.19
**Model 2**			
No	887 / 1477	1.00	Ref.
Yes (3 - <15 yo)	133 / 214	0.97	0.76–1.22
Yes (<3 yo)	107 / 182	0.93	0.72–1.20
**Model 3**			
No	887 / 1477	1.00	Ref.
Yes (3 - <15 yo)	133 / 214	0.93	0.73–1.19
Yes (<3 yo)	107 / 182	0.94	0.73–1.23

MetS: metabolic syndrome; CI: confidence interval; AOR: adjusted odds ratio; yo: years old

Model 1 adjusted for age and sex

Model 2 additionally adjusted childhood socio-economic status at 15 years

Model 3 additionally adjusted for marital status, job category, education, household income, smoking, alcohol consumption, and physical activity

Table 3 demonstrates the results of the association between parental absence by cause (death, divorce, or out-migration) and MetS. There was no significant association between parental absence by cause (i.e., death, divorce, and out-migration) during childhood and MetS in adulthood.

**Table 3:** Logistic regression analyses for the association between each cause of parental absence in childhood and metabolic syndrome in adulthood among study participants in the Khanh Hoa Cardiovascular Study, Vietnam (2019–2020).

Parental absence	Those with/without MetS	AOR	95% CI
**Due to death(s)**			
No	942 / 1579	1.00	Ref.
Yes (3 - <15 yo)	111 / 169	1.01	0.78–1.31
Yes (<3 yo)	74 / 125	0.93	0.69–1.26
**Due to divorce**			
No	1111 / 1841	1.00	Ref.
Yes (3 - <15 yo)	8 / 15	0.95	0.40–2.27
Yes (<3 yo)	8 / 17	0.79	0.33–1.85
**Due to migratory work**			
No	1070 / 1765	1.00	Ref.
Yes (3 - <15 yo)	22 / 49	0.72	0.42–1.20
Yes (<3 yo)	35 / 59	0.94	0.61–1.45

MetS: metabolic syndrome; CI: confidence interval; AOR: adjusted odds ratio; yo: years old

Model was adjusted for age, sex, and childhood socioeconomic status. The exposure variables were mutually adjusted in the model.

## Discussion

In this study, no significant associations were found between parental absence and MetS among 3,000 middle-aged community dwellers in rural Khanh Hoa Province, Vietnam. This non-significant association was also observed when specific associations related to different causes of parental absence (i.e., death, divorce, and out-migration) were examined.

The non-significant associations between parental absence and MetS did not support our hypothesis, which was based on studies by Alciati et al. [10] that reported a significant association between parental loss and MetS among severely obese subjects, and Chen et al. [9] that tended to show a significant association between parental death during childhood and increased hypertension risk during adulthood, a component of MetS. However, our findings align with a previous study conducted in China by Schooling et al. [25], which reported null association between parental death during childhood and adult cardiovascular risks such as blood pressure, fasting glucose, LDL-cholesterol and HDL-cholesterol. In addition, a few studies conducted among Western populations also reported null findings when they examined the specific association between parental divorce and marital discord (types of parental absence) and CVDs [26, 27]. In contrast to the study participants in Alciati et al.’s study [10] who were severely obese and whose parents might have been genetically susceptible to obesity or MetS, and earlier death, our study participants as well as most of the previous studies included normal population. This is one of the reasons for the discrepancy.

There are several possible interpretations of the non-significant associations. First, the participants could have also experienced stressful life events during their childhood, due to domestic turmoil during and after the Vietnam War. Hence, parental absence may not be the only strong predictor of MetS later in life. Second, those who experienced parental absence might have received care and support from extended family members, which might have lessened the negative effect of parental absence. The Vietnam War was so long that by the time the study participants were born (around 1960–1979), the adults might have been already accustomed to the conditions of war and were more likely to support children who had been separated from their parents. Third, those who were more severely affected by parental absence might have died earlier, and thus, did not participate in our study (i.e., survival bias). This supposition was supported by Brown et al. [28], who showed a significant association between ACEs and an increased risk of premature mortality among 17,337 adults in the United States.

However, it should be reminded that these interpretations are not in line with our previous finding that parental absence in childhood was significantly associated with adulthood depressive symptoms among the same participants as in this study [20]. More specifically, those who experienced parental absence at 3 - < 15 years old and before 3 years old were 1.21 times and 1.41 times more likely to have depressive symptoms, respectively. This discrepancy in the study results indicates that the impact of parental absence during childhood did have a long-lasting health effect that stretches across the life course, but it can vary depending on the health outcomes under study. Another possible interpretation is that compared to individuals in developed countries where unhealthy foods are readily available that can increase cardiometabolic risk, those in Vietnam had not lived in an environment where they could afford to excessively consume an unhealthy diet as a result of unhealthy stress coping behaviors; such a difference might have underlain the null finding observed in our study.

This study has a few limitations that should be considered while interpreting the results. First, although parental absence is unlikely to be forgotten, details (timing and length of separation with parent/s) regarding the event that had occurred in the remote past might be subject to recall bias. Second, the participants might have been hesitant to report their parents’ marital conflict (i.e., social desirability bias). Third, several variables could explain the association between parental absence and MetS, such as stressful childhood life events and the availability of social or financial support after the experience. Moreover, information on whether the experience involved losing either the mother or father or both parents could have facilitated our understanding of the impact of parental loss. Fourth, as mentioned before, the study findings might be subject to survival bias. Fifth, the number of participants with MetS might not have been sufficient to determine the associations, particularly when we examined the association in relation to parental absence caused by specific reasons; it should be mentioned that the sample size was not calculated specifically for this study. Finally, the participants might not have fully represented rural populations in Vietnam, as we only chose one district in one province in Vietnam.

## Conclusion

This study did not find any evidence of a significant association between parental absence in childhood and MetS in adulthood among middle-aged dwellers in rural Khanh Hoa Province, Vietnam. The association remained non-significant when we considered the causes and timing of parental absence.

## Supporting information

S1 File (DOCX)
